# Head Tilting Elicited by Head Turning in Three Dogs with Hypoplastic Cerebellar Nodulus and Ventral Uvula

**DOI:** 10.3389/fvets.2016.00104

**Published:** 2016-11-23

**Authors:** Shinji Tamura, Yuya Nakamoto, Takashi Uemura, Yumiko Tamura

**Affiliations:** ^1^Tamura Animal Clinic, Hiroshima, Japan; ^2^Kyoto Animal Referral Medical Center, Kyoto, Japan; ^3^Department of Neurology, Japan Animal Referral Medical Center, Kawasaki, Japan; ^4^Department of Bioartificial Organs, Institute for Frontier Medical Sciences, Kyoto University, Kyoto, Japan

**Keywords:** cerebellar, nodulus, ventral uvula, dog, positioning head tilt

## Abstract

The nodulus and ventral uvula (NU) of the cerebellum play a major role in vestibular function in humans and experimental animals; however, there is almost no information about NU function in the veterinary clinical literature. In this report, we describe three canine cases diagnosed with presumptive NU hypoplasia. Of them, one adult dog presented with cervical intervertebral disk disease, and two juvenile dogs presented with signs of central vestibular disease. Interestingly, an unusual and possibly overlooked neurological sign that we called “positioning head tilt” was observed in these dogs. The dogs were able to turn freely in any direction at will. The head was in a level position when static or when the dog walked in a straight line. However, the head was tilted to the opposite side when the dog turned. Veterinary clinicians should be aware of this neurological sign that has not been reported previously, and its application in lesion localization in dogs.

## Introduction

The vestibulo-cerebellum is described in the veterinary literature as consisting of the flocculonodular lobe (flocculus and nodulus) and the fastigial nuclei and as being principally responsible for maintenance of equilibrium and coordination of head and eye movements (1–5). In humans and experimental animals, however, the vestibulo-cerebellum consists of the flocculus and ventral paraflocculus that are involved in vestibulo-ocular plasticity and smooth pursuit eye movements, as well as the nodulus and ventral uvula (NU) that are thought to be involved in spatial orientation (6–10). NU lesions in humans result in severe spatial disorientation, postural instability, spontaneous nystagmus, vomiting, and marked deficits in the vestibulo-ocular reflex. These effects indicate that the NU play a major role in vestibular function, although the role of each part of the vestibulo-cerebellum is not clearly understood (11). To date, there is almost no information about NU function in the veterinary clinical literature.

In this report, we present three cases of canines with presumptive NU hypoplasia and resultant head tilting in response to head movement caused by lack of inhibition of the vestibular nuclei by the NU. We name this symptom “positioning head tilt.”

## Case Presentation

### Case 1

**Figure 1:**
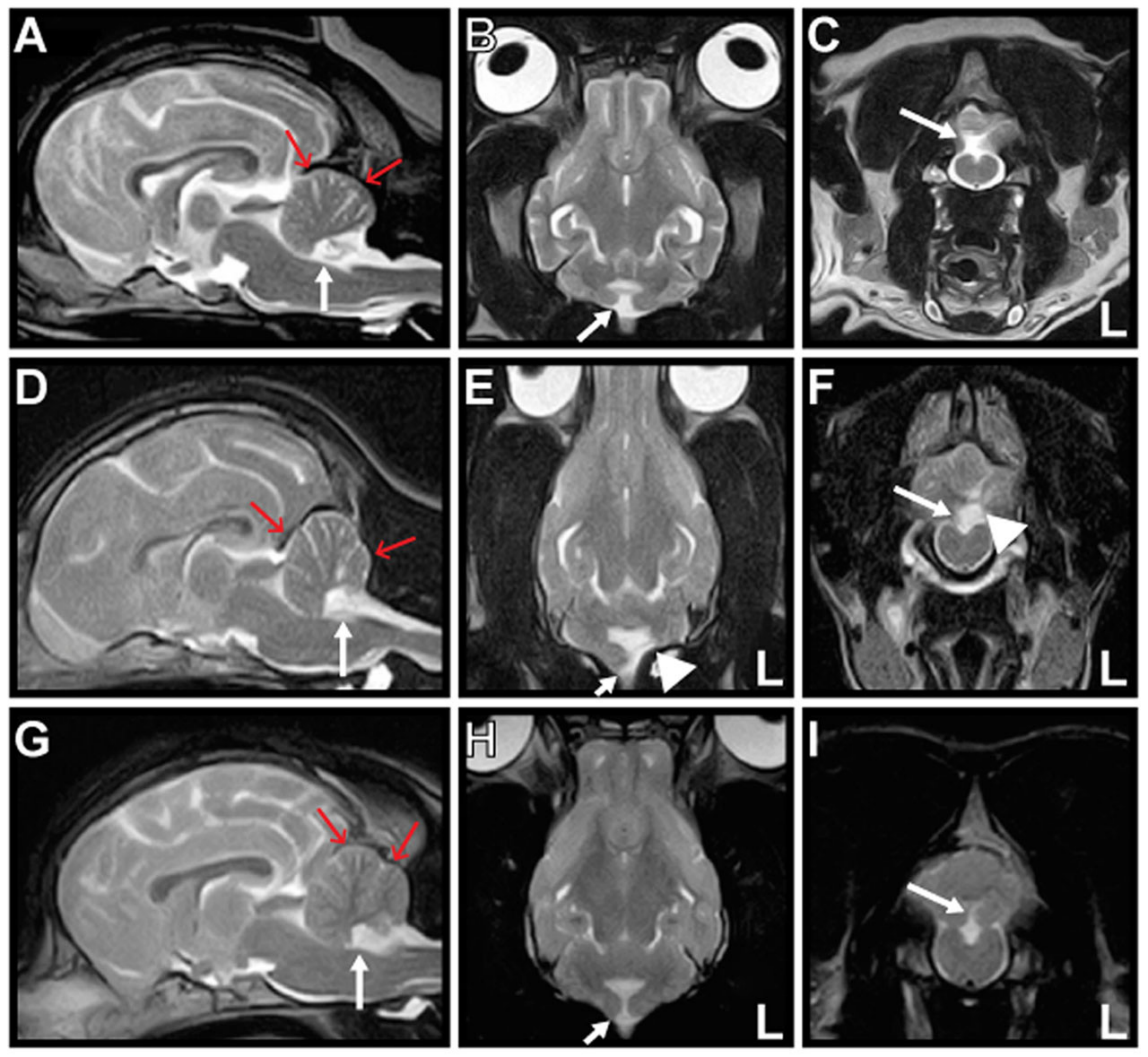
**Magnetic resonance T2-weighted images of Cases 1–3**. Mid-sagittal **(A,D,G)**, dorsal **(B,E,H)**, and transverse **(C,F,I)** images are shown. The nodulus and ventral uvula of the cerebellum (arrow) are absent in all cases. The caudal fossa (red arrows) is reduced in size, indicating hypoplasia in all cases. There is also partial absence of the left cerebellar hemisphere (arrowhead) in Case 2 **(E,F)**.

A 10-year-old neutered-male Pug dog presented to Tamura Animal Clinic in 2013 with a history of acute tetraparesis. A general physical examination revealed posterior synechiae in the left eye and no abnormalities on palpation and auscultation. On neurological examination, in addition to ambulatory tetraparesis, head tilting without a fixed direction was observed. It was unclear how long this head tilt had been present, as the owner had not noticed it previously. The dog had not exhibited any previous neurological episodes that might have been related to this sign. The complete blood count and serum biochemistry results were mostly within the reference intervals (RIs). An antigen test for *Dirofilaria immitis* was negative. Thoracic and abdominal radiographs revealed no abnormality. Magnetic resonance imaging (MRI) and radiography of the cervical spine performed on the initial presentation day revealed that the tetraparesis was caused by C3–4 intervertebral disk disease that was treated surgically using a ventral slot decompression procedure on the same day. After the tetraparesis had resolved a week after surgery, the head tilt was still present and reevaluated during observation of free walking. The dog could walk freely in any direction at will. The head was in a level position when static or when the dog was walking in a straight line. However, the head was consistently tilted to the right when the dog turned to the left, and to the left when the dog turned to the right (Video S1 in Supplementary Material). We called this neurological sign “positioning head tilt.” Physiological nystagmus was present, spontaneous or positional nystagmus was not observed in any head position, and other neurological signs were not observed. Neurolocalization to the vestibular system was determined. Brain MRI (0.3-T AIRIS2 Comfort; Hitachi Medical Co., Tokyo, Japan) had been performed during evaluation for cervical intervertebral disk disease on the initial presentation day. T2-weighted [T2W; repetition time (TR) (ms)/echo time (TE) (ms), 4000/120] sagittal, coronal, and transverse images; T2W fluid-attenuated inversion recovery (FLAIR) (TR/TE, 8500/120) transverse images; and T1-weighted (T1W; TR/TE, 400/15) transverse images were acquired. Post-contrast T1W sagittal, coronal, and transverse images were acquired after intravenous administration of gadopentetate dimeglumine (Magnevist; Bayer, Tokyo, Japan; 0.15 mmol/kg). The NU of the cerebellum were absent in all sequences. Reduction in size of the caudal fossa was also evident in mid-sagittal images. There was no other abnormality, and no abnormal contrast enhancement was observed in the brain (Figures 1A–C). Hypoplasia of the caudal fossa was suspected. These findings were most likely consistent with NU hypoplasia. Analysis of cerebrospinal fluid was not performed. The positioning head tilt was not treated and was still present when the dog was reexamined at our hospital 2 years later.

### Case 2

A 5-month-old female Shiba dog presented to Bingo Pet Clinic (Fukuyama City, Hiroshima Prefecture) in 2015 with a history of circling. Since the owner had acquired the dog at 2 months of age, it had exhibited throwing of the head back and forth when looking into the distance. Peracute onset of rolling to the left was observed before the dog was brought to the clinic, but this symptom disappeared within several minutes. Although abnormal rolling or circling was not observed at presentation, slight ataxia and head tilt to either side were identified, and the dog was referred to Tamura Animal Clinic 10 days after the initial consultation. Positioning head tilt was observed during observation of free walking. Most of the time, but not always, the head was tilted in response to head movement. Positioning head tilt had been observed by the owner since acquiring the dog, but the owner had considered this head tilt a peculiarity. Ataxia of the whole body was observed when the dog shook her head or scratched her ear with a hind limb (Video S2 in Supplementary Material). General physical examination including palpation and auscultation revealed no abnormality. On neurological examination, decreased menace response with normal pupillary light reflexes and normal vision was observed. Physiological nystagmus was present, and there was no spontaneous or positional nystagmus in any head position or any other neurological abnormality. Neurolocalization to the cerebellum was determined. The complete blood count and serum biochemistry results were mostly within the RIs. Radiography and ultrasonography of the thorax and abdomen, urinalysis, and fecal examination revealed no abnormality. Brain MRI (0.3-T AIRIS2 Comfort; Hitachi Medical Co., Tokyo, Japan) was performed on the initial presentation day. T2W (TR/TE, 4000/120) sagittal, coronal, and transverse images; FLAIR (TR/TE, 8500/120) sagittal and transverse images; and T1W (TR/TE, 400/15) transverse images were acquired. Post-contrast T1W sagittal, coronal, and transverse images were acquired after IV administration of gadopentetate dimeglumine (Magnevist; Bayer, Tokyo, Japan; 0.15 mmol/kg). Absence of the NU and a small part of the left cerebellar hemisphere with hypoplasia of the caudal fossa was revealed. There was no other abnormality, and no abnormal contrast enhancement was observed in the brain (Figures 1D–F). These findings were most likely consistent with NU hypoplasia and partial hypoplasia of the left cerebellar hemisphere. Analysis of cerebrospinal fluid was not performed. No treatment was administered for the positioning head tilt. The positioning head tilt had improved but had not disappeared, and the ataxia had disappeared, when the dog was 1 year of age. The menace response was not evaluated at that time.

### Case 3

A 9-month-old female French bulldog was presented to Kyoto Animal Referral Medical Center in 2015 with a history of head tilt with no fixed direction. Peracute right-sided head tilt and horizontal nystagmus to the left were observed 1 week before the dog was examined by us, but these symptoms had disappeared in several minutes. Positioning head tilt was observed during observation of free walking. Some of the time, but not always, the head was tilted in response to head movement. The owner had recognized this head tilt as a peculiarity, and the symptom had been present from at least 2 months of age. General physical examination including palpation and auscultation revealed no abnormality. On neurological examination, physiological nystagmus was present, and spontaneous or positional nystagmus was not observed in any head position. When the dog started to walk, a spastic gait was observed in the right hind limb. A slight decrease in conscious proprioception was present in both hind limbs. Muscle tone, pelvic limbs spinal reflexes, and cutaneous trunk reflex were all normal, and spinal pain was not observed. The dog suddenly showed left-sided head tilt and horizontal nystagmus to the right during the consultation; however, these symptoms disappeared in several minutes. Neurolocalization to the vestibular system and T3–L3 spinal cord was determined. The complete blood count and serum biochemistry results were mostly within the RIs. Thoracic and abdominal radiography revealed hemivertebrae in the thoracic spine. Electrocardiography and echocardiography revealed no abnormal findings. Brain MRI (0.3-T AIRIS Vento; Hitachi Medical Co., Tokyo, Japan) was performed on the initial presentation day. T2W (TR/TE, 4000/100) sagittal, coronal, and transverse images; FLAIR (TR/TE, 9000/100) sagittal and transverse images; and T1W (TR/TE, 380/15) sagittal and transverse images were acquired. Post-contrast T1W sagittal and transverse images were acquired after intravenous administration of gadopentetate dimeglumine (Magnevist; Bayer, Tokyo, Japan; 0.15 mmol/kg). Absence of the NU and hypoplasia of the caudal fossa were revealed. There were no other abnormalities, and no abnormal contrast enhancement was observed in the brain (Figures 1G–I). These findings were most likely consistent with NU hypoplasia. Analysis of cerebrospinal fluid was not performed. Although obvious compression of the spinal cord was not observed on spinal MRI, the decreased conscious proprioception in both hind limbs was suspected to be associated with the hemivertebrae. No treatment was administered for the “positioning head tilt.” Although no further peracute vestibular episodes were observed after consultation with us, the positioning head tilt was still present 15 months later.

## Discussion

Cerebellar hypoplasias involving the NU in dogs have often been reported as cerebellar vermian hypoplasia or Dandy–Walker syndrome (also known as Dandy–Walker malformation complex) (12–16). It is difficult to identify missing parts of canine vermis on MRI, especially with low field MRI. This explains the previously used term cerebellar vermian hypoplasia to describe a lack of any part of the vermis. These dogs are usually euthanized at several weeks to months of age because of marked neurological dysfunction such as severe ataxia, tremor, and hypermetria. Dandy–Walker malformation in humans is usually characterized by an absent or small cerebellar vermis, cystic malformation of the fourth ventricle, and enlargement of the posterior fossa (17), while cystic malformation of the fourth ventricle without enlargement of the posterior fossa is referred to as Dandy–Walker variant (18, 19). The affected areas in our cases were fewer than those of previously reported canine cases of cerebellar hypoplasia, similar to Dandy–Walker variant in human patients, and the missing and remaining part of the vermis were able to be identified. In addition, the neurological signs in our cases were much more minor than that of previously reported canine cases (12–16). Positioning head tilt had not been noticed by the owners in Case 1 and only been considered a peculiarity in Cases 2 and 3, i.e., until they were identified by detailed observation and neurological examination in a veterinary clinic. This sign therefore seems to be difficult to recognize as a clinical symptom by both owners and veterinarians because it is minor and does not disturb quality of life. This difficulty of recognition may be the reason why the positioning head tilt has not been reported in the veterinary literature.

The positioning head tilt observed in our cases may be explained by the following hypothesis. When the head is static or when the subject walks in a straight line, bilateral vestibular nuclei are equally stimulated by both vestibular organs, and the head is maintained in a level position. Vestibular nuclear projections to the spinal cord are situated in the vestibulospinal tracts. The fibers terminate on interneurons that facilitate ipsilateral flexors and decussate to inhibit contralateral extensor muscle activity. The vestibulospinal tracts facilitate spinal reflexes, especially those involved in maintaining posture and the antigravity/extensor muscles. Turning the head to the left causes a shift in the distribution of body mass to the left. This shift in mass is supported by increased extension on the left side, which is reflexively induced by both myotatic and vestibular reflexes. This also reduces extension on the right side, and thereby minimizes weight transfer to the left (20). The NU coordinate this system by inhibition of stimulation of vestibular nuclei in order to maintain a level head position in response to head movement, and this inhibition was absent in our cases. As a result, the head was tilted to the opposite side from the turning direction in these dogs. Compensation for positioning head tilt may occur with time, as was observed in Case 2.

A congenital origin for the abnormal NU is more likely than a lesion secondary to infarction of a branch of the bilateral caudal cerebellar arteries because of the presence of hypoplasia of the caudal fossa and no history of the dog having had any episode of a suspected cerebellar vascular accident in Case 1, and positioning head tilt had been observed before the peracute vestibular episode in Cases 2 and 3.

The causes of the ataxia and decreased menace response observed in Case 2 and peracute episodic vestibular signs observed in Cases 2 and 3 were undetermined. Another disease process (inflammatory, infectious, metabolic, or toxic) cannot be ruled out because of the lack of CSF analysis. However, these signs might have been specific to the acute phase of NU dysfunction, because they were seen in the juvenile dogs and later disappeared. The partial hypoplasia of the left cerebellar hemisphere in Case 2 might also have contributed to these signs. It is interesting that the direction of the vestibular signs differed between the 2 episodes in Case 3. This may have been due to bilateral NU lesions.

Some genetic mutations have been identified in humans with cerebellar vermis hypoplasia (21). There is no information about the genetics of canine cases, and DNA tests were not performed in our cases. DNA tests may make it possible to differentiate congenital malformation from another disease process in the future.

Clinical findings in humans with lesions affecting the NU have been reported previously (22) and include the following: prolongation of vestibulo-ocular responses (increased velocity storage); loss of ability to suppress post-rotational nystagmus by tilting the head when the rotation stops; positional nystagmus (downbeat nystagmus); and periodic alternating nystagmus in darkness (present in light if flocculus and paraflocculus are also affected, which impairs visual function). The first and second signs in this list were not evaluated in our cases because we did not have the clinical methods to evaluate them, e.g., velocity of physiological nystagmus (vestibulo-ocular responses) in veterinary medicine. Although physiological nystagmus was observed in our cases, it was unclear whether this was normal. This is because diagnostic criteria for evaluating eye movement during physiological nystagmus remain has not been clarified in veterinary medicine. Positional nystagmus was not observed, and eye movement in darkness was not evaluated, in the present cases.

Limitations of this study include the small number of cases and the lack of pathological diagnoses in all cases. However, the locations of focal lesions were clearly identified on MRI in all dogs, so it is appropriate to discuss the relationship between neurological signs and lesion localization. Detailed observation of the clinical signs and course in large numbers of cases with similar characteristics to our cases, as well as pathological diagnosis, is warranted.

## Concluding Remarks

This novel information about the neurological signs in canine NU disorder may help veterinary clinicians to localize the lesion in dogs with this condition, as well as neurophysiologists to acquire further understanding of the functions of the NU.

## Consent

Consent procedure is not applicable, as this article is a retrospective review of clinical cases.

## Author Contributions

All the authors met the criteria for authorship. ST, YN, TU, and YT participated in clinical case management. ST drafted the manuscript. ST and YN reviewed the neuroimaging studies. ST, YN, and YT participated in the review and the editing of the manuscript.

## Conflict of Interest Statement

The authors declare that the research was conducted in the absence of any commercial or financial relationships that could be construed as a potential conflict of interest.

## Supplementary Material

Video S1 **Positioning head tilt observed in Case 1**.

Video S2 **Positioning head tilt and ataxia observed in Case 2**.
